# Needle Fracture during Endoscopic Ultrasound-Guided Fine-Needle Aspiration of Suspicious Thoracic Lymph Nodes

**DOI:** 10.1155/2016/2526789

**Published:** 2016-07-31

**Authors:** Bartosz Adamowicz, Thibaut Manière, Vincent Déry, Étienne Désilets

**Affiliations:** Charles-Lemoyne Hospital, Medicine and Gastroenterology Department, Université de Sherbrooke, 3120 Boulevard Taschereau, Greenfield Park, QC, Canada J4V 2H1

## Abstract

Endoscopic ultrasound fine-needle aspiration (EUS-FNA) is used to make a cytopathologic diagnosis of suspicious lesions located around the gastrointestinal tract. It is a safe technique with few complications. The most common complications of EUS-FNA are related to pancreatic lesions (pancreatitis, bleeding, and abdominal pain). Rare complications have been noted such as stent malfunction, air embolism, infection, neural and vascular injuries, and tumor cell seeding. There are very few studies examining equipment malfunctions. We report a case of needle fracture during the EUS-FNA of suspicious thoracic lymph nodes in a 79-year-old man investigated for unexplained weight loss.

## 1. Introduction

Endoscopic ultrasound fine-needle aspiration (EUS-FNA) is used to make a cytopathologic diagnosis of suspicious lesions located around the gastrointestinal tract. These include mediastinal lymph nodes easily accessible with the transesophageal position. It is a safe technique with few complications. A recent review article [1] found an EUS-FNA-specific morbidity of 0.98% and a mortality of 0.02%. The most common complications of EUS-FNA are related to pancreatic lesions (pancreatitis, bleeding, and abdominal pain). Rare complications have been noted such as stent malfunction, air embolism, infection, neural and vascular injuries, and tumor cell seeding [1–3]. There are very few studies examining equipment malfunctions.

## 2. Case Report

A 79-year-old man, past smoker, known for hypertension and diabetes, was hospitalized for an unexplained weight loss. During the initial investigation, multiple lymphadenopathies were discovered on a thoracic scanner including lymph nodes located anterior to the trachea (2.1 × 2.5 cm), in the aortopulmonary window (1.5 × 3.5 cm), inferior to the carina (1.5 × 3.5 cm), and at the right and left hilum measuring, respectively, 3.5 × 2.7 cm and 3 × 1.2 cm. In addition, peribronchial micronodules were located in the right and left superior lobes, the inferior right lobe, and the root of the middle lobe. A bronchoscopy was performed. Reactive cellular changes without malign neoplastic cells were found on the bronchial washing, while the bronchial biopsy demonstrated nonnecrotizing granuloma inflammation. Multiple possible diagnoses were retained including lymphoma, non-small cell pulmonary neoplasm, sarcoidosis, and tuberculosis.

**Figure 1:**
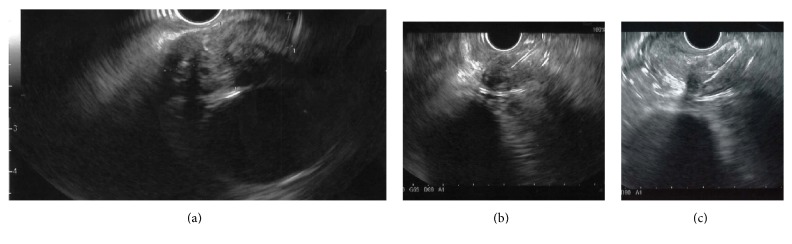
Subcarinal lymphadenopathy on transesophageal EUS (a). EUS-FNA attempt with a 19-gauge needle (b). EUS-FNA attempt with a 22-gauge needle (c).

**Figure 2:**
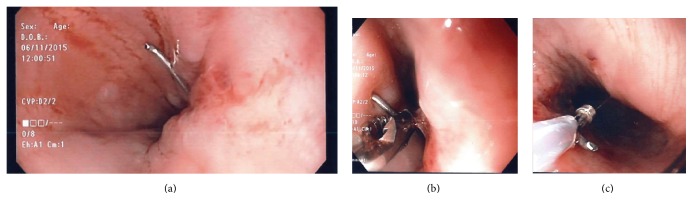
The fractured EUS needle visible in the esophageal lumen with a gastroscope (a). Multiple removal attempts with an alligator forceps (b) and an extraction basket (c).

**Figure 3:**
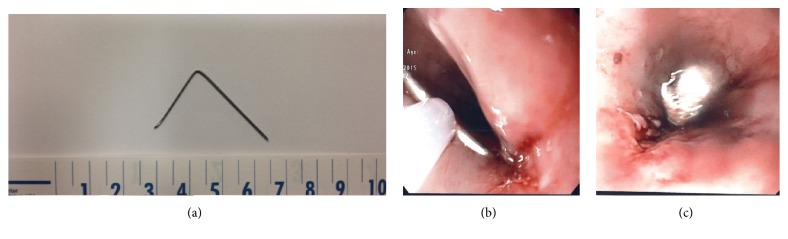
The extracted fractured needle (a). Notice the bent V shape made on the needle with the polypectomy snare and the overtube that permitted the extraction (b). Endoscopic clip on the puncture site (c).

Under conscious sedation, an EUS was performed to biopsy a 30 × 15 mm, partially calcified, hypoechogenic subcarinal lymph node (GF-UCT180, Olympus Medical Systems, Center Valley, Pa) [Figure 1(a)]. During the procedure, an initial attempt was made to gather material using the 19-gauge needle (Expect*™* Slimline Needle, Boston Scientific) [Figure 1(b)]. An inadequate amount of tissue was extracted due to the rigidity of the lymph node. A second attempt was made using the 22-gauge needle (Expect*™* Slimline Needle, Boston Scientific) [Figure 1(c)]. Significant rigidity was again noted during puncture but the needle made it through the lymph node twice. The fanning technique with repeated erector movement was tried during the first and second passage but failed to adequately move the needle given the hard texture of the lymph node. The needle fractured during removal after the second passage as the operator was using significant force on the handle and torque on the endoscope feeling that the needle was stuck in the lymph node. The distal part of the needle was visible, pinned in the esophagus wall, while the proximal part remained attached to the needle sheet [Figure 2(a)]. After switching to a standard gastroscope (GIF-HQ190, Olympus Medical Systems), an esophageal overtube was installed and multiple attempts were made to remove the needle using various tools, including a polypectomy snare, an alligator jaw forceps [Figure 2(b)], and an endoscopic basket [Figure 2(c)]. The tip of the needle seemed to have stayed in the rigid lymph node since all instruments were sliding on the needle even with good grip and significant traction force. After numerous tries, the fractured needle was grabbed with a polypectomy snare (sensation oval 27 mm, Boston Scientific) which hardly closed on the needle near the esophageal wall [Figure 3(b)]. The needle was then bent using the overtube by pushing the tube downward and by forcing the needle upward in the tube. With the bent needle now in a V shape, we were able to keep the closed polypectomy snare at the bottom of the V and apply a good traction force to extract the needle [Figure 3(a)]. Two endoscopic clips were used to close the puncture site [Figure 3(c)].

**Figure 4:**
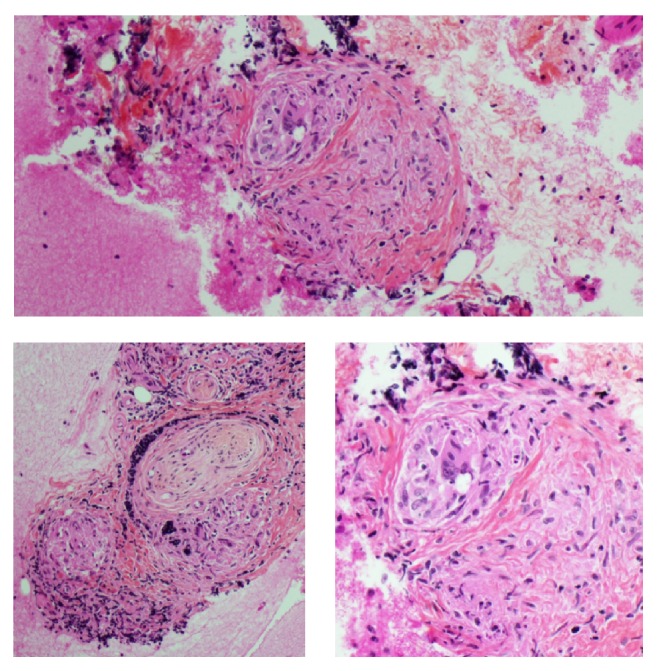
Histology from fine-needle aspiration showing nonnecrotizing granulomatous inflammation.

A thoracic scan following the procedure showed a mild paraesophageal pneumomediastinum and a discreet 29 × 4 mm parietal hematoma, but no signs of perforation with ingestion of contrast. The patient was completely asymptomatic and did not require any surgical intervention. A control CT scan showed no oral contrast extravasation and complete resolution of the pneumomediastinum within 24 hours. The patient was then able to eat without sequelae. The biopsy specimen demonstrated a few small nonnecrotizing granulomas, epithelioid histiocytes, and rare giant cells. An infectious process or sarcoidosis was retained as possible diagnosis [Figure 4].

## 3. Discussion

Many authors have explored the complications associated with EUS-FNA [1–3], but only a few have explored needle malfunctions specifically.

In their study, comparing the effectiveness of the 22-gauge and 25-gauge needles in the diagnosis of pancreatic solid mass, Siddiqui et al. [4] mentioned that there were 16 cases of malfunctioning equipment due to bending needles or loss of handle maneuverability without any needle fracture. In a study comparing 22-gauge aspiration and 22-gauge biopsy needles in pancreatic cancer, only 1 technical failure was found due to a detached stylet cap [5]. DeWitt et al. [6] experienced a needle fracture while performing a transenteric pancreatic duct stenting for a patient with pancreaticojejunal anastomosis following pancreaticoduodenectomy. The authors suspected that a combination of factors contributed to the needle breaking, including endoscopic techniques, mainly torqueing and multiple punctures, and the rigid texture of the pancreas. Similarly, our patient had very rigid lymph node that may have also contributed to the break in the needle.

There is more literature on needle fractures in pulmonary medicine. In fact, a recent survey examining complications associated with endobronchial ultrasound-guided transbronchial needle aspiration (EBUS-TBNA) done by the Japan Society for Respiratory Endoscopy [7] found needle fracture in 15 out of 7,345 procedures. Recently, Vial et al. [8] also experienced a similar problem, when a needle fractured during an EBUS-guided TBNA needle aspiration of a left paratracheal lymphadenopathy. The authors did not mention whether endoscopic maneuvers or the quality of the lymph nodes might have played a role. After multiple removal attempts, it was decided to leave the needle in place without surgery. No information was provided whether further complications were experienced as a result of the foreign body being imbedded in the lymph node. Similarly, during the EBUS-TBNA of a subcarinal lymph node, Özgül et al. [9] experienced the breaking of a 22-gauge needle after the third passage, leaving an 11 mm fragment in the airways. The authors noted that the texture of the lymph nodes was normal and they did not have difficulties in reaching them. A manufacturing error was suspected. The needle could not be retrieved on the subsequent urgent bronchoscopy, as it was no longer in the airways. It was later located in the transverse colon on an abdominal X-ray. The patient remained asymptomatic and no complications were observed on follow-up. Dhillon and Yendamuri [10] also had a comparable experience during an EBUS-guided TBNA for suspicious mediastinal lymphadenopathy. The needle broke from its sheath and protruded outwards. Fortunately, the broken needle was still attached to the rest of the apparatus and was thus removed in one piece without any remnants left in the patient. Once again, standard endoscopic techniques were used and there was only a single aspiration done. The texture of the tissue was not described.

Presently, the consequences of a broken needle during ultrasound-assisted techniques are unknown due to the limited literature on the subject. It is possible to assume that if the foreign body is left in situ, an inflammatory reaction may develop within the tissue leading to further complications. In addition, there is always the possibility of the fragments dislodging and migrating while damaging the mucosal lining. This may result in perforation and surgical intervention. It is difficult to identify specific risk factors for needle fracture. However, as mentioned by other authors, certain endoscopic maneuvers as torqueing, multiple punctures with the same needle, and rigid tissue texture could all be predisposing factors. We also feel that repeated erector movement for the fanning technique in the hard lymph node may have caused a focal weakness on the needle in our case. It is important for clinicians to be aware not only of complications associated with ultrasound-guided techniques, but also of complications related to the devices themselves including fractured needles.
